# PySFD: comprehensive molecular insights from significant feature differences detected among many simulated ensembles

**DOI:** 10.1093/bioinformatics/bty818

**Published:** 2018-09-21

**Authors:** Sebastian Stolzenberg

**Affiliations:** Department of Mathematics and Computer Science, Computational Molecular Biology Group, Arnimallee 6, 14195 Berlin, Germany

## Abstract

**Motivation:**

Many modeling analyses of molecular dynamics (MD) simulations are based on a definition of states that can be (groups of) clusters of simulation frames in a feature space composed of molecular coordinates. With increasing dimension of this feature space (due to the increasing size or complexity of a simulated molecule), it becomes very difficult to cluster the underlying MD data and estimate a statistically robust model. To mitigate this “curse of dimensionality”, one can reduce the feature space, e.g., with principal component or time-lagged independent component analysis transformations, focusing the analysis on the most important modes of transitions. In practice, however, all these reduction strategies may neglect important molecular details that are susceptible to experimental verification.

**Results:**

To recover such molecular details, I have developed PySFD (Significant Feature Differences analyzer for Python), a multi-processing software package that efficiently selects significantly different features of any user-defined feature type among potentially many different simulated state ensembles, such as meta-stable states of a Markov State Model (MSM). Applying PySFD on MSMs of an aggregate of 300 microseconds MD simulations recently performed on the major histocompatibility complex class II (MHCII) protein, I demonstrate how this toolkit can extract and visualize valuable mechanistic information from big MD simulation data, e.g., in form of networks of dynamic interaction changes connecting functionally relevant sites of a protein complex.

**Availability and implementation:**

PySFD is freely available under the L-GPL license at https://github.com/markovmodel/PySFD.

**Supplementary information:**

Supplementary data are available at *Bioinformatics* online.

## 1 Introduction

Fueled by perpetual advances in supercomputing capabilities (Glaser *et al.*, 2015; Shaw *et al.*, 2009; Stone *et al.*, 2010), high-throughput molecular dynamics (MD) simulations of increasing size and timescales are becoming amenable. Most often, these advances are paralleled with an increasing heterogeneity in supercomputing resources (e.g., different compute nodes containing different numbers and types of CPUs/GPUs). This heterogeneity is reflected in high-throughput MD datasets in terms of numbers and lengths of individual MD simulations, and thus demands means to analyze these data appropriately (Faradjian and Elber, 2004; Preto and Clementi, 2014; Schaudinnus *et al.*, 2016; Wriggers *et al.*, 2009): For example, Markov State Models (MSMs; Bowman *et al.*, 2014; Noé *et al.*, 2009) are capable to compute thermodynamic and kinetic properties from many shorter off-equilibrium MD simulations, which for practical supercomputing reasons (inter-core/-node communication times, job queuing policies) are much more feasible to generate than a single, or a few long MD simulations. Also, MSMs can be used “on-the-fly” in adaptive MD simulations (Bowman *et al.*, 2010; Doerr and De Fabritiis, 2014; Plattner and Noé, 2015) that continuously select new restarting points to enhance the sampling of under-explored molecular conformations or transitions.

In particular, MSMs estimate transition probabilities between micro-states, which are usually defined as clusters in a conformational feature space (inter-atomic distances, backbone dihedrals, chain rotamers, …). The more complex a simulated system, the higher the dimension of this feature space, which requires more and better clusters, and more observed inter-cluster transitions to estimate a statistically robust MSM. This “curse of dimensionality” for such feature spaces can be alleviated by including only coarse-grained or fewer localized features, and/or only the most important, uncorrelated eigen modes in such feature space (principal component analysis, or time-lagged independent component analysis) (Pérez-Hernández *et al.*, 2013; Pérez-Hernández and Noé, 2016). By definition, all these reduction strategies (and thus an MSM) do not encode all molecular features at the same time, many of which may be equally important to understand a protein’s mechanism. In principle, however, such important features may be recovered a posteriori because each MSM micro-state represents a set of simulation frames, and thus an average feature value. For example, by correlating average feature values with MSM eigenvectors along micro-state (Pérez-Hernández *et al.*, 2013), one can thus extract features that represent best the slowest eigenvectors of an MSM. Alternatively, one can identify significant feature differences (SFDs) among pairs of simulated ensembles—e.g., meta-stable states (sets) of micro-states, even across different mutants—as implemented for non-covalent contact frequencies in (Farabella *et al.*, 2014), or in the PIA (Stolzenberg, 2014; Stolzenberg *et al.*, 2015, 2016), and pyHVis3D (Knapp *et al.*, 2018) tools. In this paper, I have developed the object-oriented Python package PySFD (Significant Feature Differences analyzer for Python), a generalized and more powerful framework that efficiently detects and visualizes significant differences in any user-defined feature between many pairs or many groups of molecular simulation state ensembles. As a result, these significantly different features can be used to distinguish or even classify these ensembles from one another for verification of stationary distributions (estimated, e.g., from MSMs) and their underlying simulations, and further inspire novel molecular predictions that are directly testable in experiments, such as mutagenesis or substituted cysteine accessibility measurements (Liapakis *et al.*, 1999).

In this paper, I describe the basic concepts of PySFD, and its main functionalities. In the Supplementary Information, I illustrate its capabilities by applying it on 300*μ*s MD simulations I had performed on an MHCII (HLA-B1DR*01:01) protein complex (Wieczorek *et al.*, 2016), an important peptide exchanger in the adaptive immune system.

## 2 Materials and methods

**Fig. 1.**
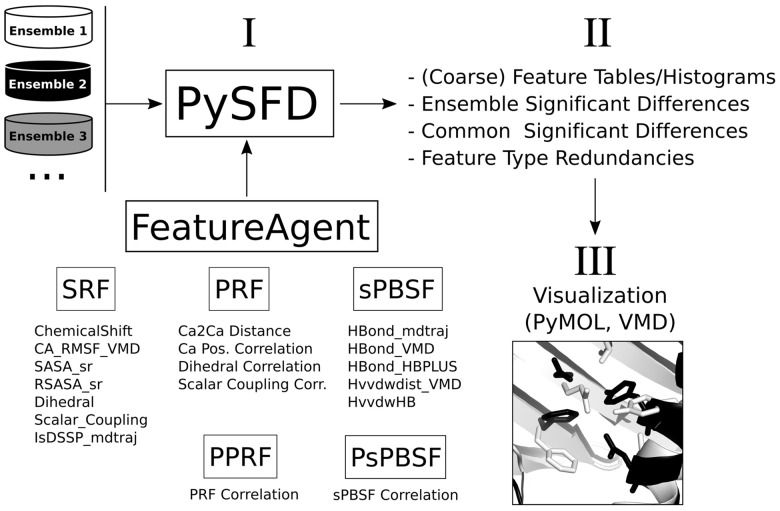
Workflow of the PySFD software: The *PySFD* main class receives input trajectories realizing different molecular ensembles, and *FeatureAgent*-derived classes (see Supplementary Methods section), i.e. SRF (single residual feature), PRF (pairwise residual feature), sPBSF (sparse pairwise backbone/side-chain feature), PPRF (pairwise, pairwise residual feature), and PsPBSF (pairwise sparse pairwise backbone/side-chain feature) as arguments (I) to compute (coarse-grained) feature tables and histograms, feature type redundancies, and (common) significant feature differences (SFDs) among the simulated ensembles (II). The feature difference tables can be visualized via PyMOL and/or VMD (III), as illustrated in the lower right corner by white and black ribbons representing snapshots of the simulated ensembles 1 and 2, respectively. Residues with SFDs (here, *χ*_1_ rotamers) are rendered as sticks and colored by their corresponding ensemble

Given a number of molecular input trajectories for each simulated state ensemble, PySFD detects and visualizes SFDs in three different stages (I-III, Fig. 1): In the Feature Extraction stage (I), PySFD considers various groups of feature types (*SRF*, *PRF*, *sPBSF*, *PPRF*, *PsPBSF*, see Supplementary Information) in form of classes inherited from the *FeatureAgent* class. In each simulation frame, features are tabulated as Python pandas data frames (McKinney, 2010) and can be further coarse-grained into user-defined regions by residual identity (and optionally by backbone/side-chain identity) via a user-defined function (e.g., mean or sum). In stage II (see Supplementary Information), these feature tables are aggregated into means (and optionally higher statistical moments) with uncertainties, providing a way to characterize different state ensembles and/or simulated systems in form of SFDs. These differences can then be used to study molecular mechanisms directly, or to generate state- and/or system-independent insights, e.g., in form of feature type redundancies or feature selection input for various machine/deep learning algorithms. In stage III (see Supplementary Information), these SFDs can be overlaid with molecular representations of the simulated system using the PyMOL (Schrödinger, 2010) or VMD (Humphrey *et al.*, 1996) programs. In any of these cases, it remains the user’s responsibility to choose/define meaningful feature types and other PySFD parameters, and interpret the results in accordance with the particular scientific question being addressed.

## 3 Conclusion

PySFD is an object-oriented Python package I have developed to detect and visualize significant feature differences among molecular simulations, such as MD ensembles. In the Supplementary Information, I have applied PySFD on meta-stable MSM sets of 300 microseconds of MD simulation performed on the MHCII protein complex. From a machine/deep learning perspective, PySFD selects (i.e. “filters”) features that are significantly different between simulated ensembles (i.e. “classes”). This selection strategy is similar to the feature selection with one-way analysis of variance (ANOVA) (Saeys *et al.*, 2007), which performs F-tests between inter-ensemble and intra-ensemble variances, and which is directly accessible to PySFD feature tables, e.g., via the *scikit-learn* Python package (Pedregosa *et al.*, 2011). However, PySFD differs from “ANOVA” as it retains information about the sign and magnitude of each individual SFD, which makes PySFD’s “pre-learning” analysis by itself very useful.

## Supplementary Material

Supplementary Data

## References

[bty818-B1] Bowman G.R. et al (2010) Enhanced modeling via network theory: adaptive sampling of markov state models. J. Chem. Theory Comput., 6, 787–794. 23626502 10.1021/ct900620b PMC3637129

[bty818-B2] Bowman G.R. et al (2014) An Introduction to Markov State Models and Their Application to Long Timescale Molecular Simulation, Vol. 797. Springer Science & Business Media, New York.

[bty818-B3] Doerr S., De Fabritiis G. (2014) On-the-fly learning and sampling of ligand binding by high-throughput molecular simulations. J. Chem. Theory Comput., 10, 2064–2069. 26580533 10.1021/ct400919u

[bty818-B4] Farabella I. et al (2014) Allosteric signalling in the outer membrane translocation domain of papc usher. Elife, 3, 79–91. 10.7554/eLife.03532 PMC4356140 25271373

[bty818-B5] Faradjian A.K., Elber R. (2004) Computing time scales from reaction coordinates by milestoningx. J. Chem. Phys., 120, 10880–10889. 15268118 10.1063/1.1738640

[bty818-B6] Glaser J. et al (2015) Strong scaling of general-purpose molecular dynamics simulations on gpus. Comp. Phys. Commun., 192, 97–107.

[bty818-B7] Humphrey W. et al (1996) Vmd: visual molecular dynamics. J. Mol. Graph., 14, 33–38. 8744570 10.1016/0263-7855(96)00018-5

[bty818-B8] Knapp B. et al (2018) pyhvis3d: visualising molecular simulation deduced h-bond networks in 3d: application to t-cell receptor interactions. Bioinformatics, 1, 3. 10.1093/bioinformatics/btx842 29329361

[bty818-B9] Liapakis G. et al (1999) The substituted-cysteine accessibility method (scam) to elucidate membrane protein structure. Curr. Protocols Neurosci., 8, 4–15. 10.1002/0471142301.ns0415s08 18428478

[bty818-B10] McKinney W. (2010) Data structures for statistical computing in python In: van der Walt S., Millman J. (eds), Proceedings of the 9th Python in Science Conference, pp. 51–56. https://scholar.google.de/scholar?q=data+structures+for+statistical+computing+in+python&hl=en&as_sdt=0&as_vis=1&oi=scholart.

[bty818-B11] Noé F. et al (2009) Constructing the equilibrium ensemble of folding pathways from short off-equilibrium simulations. Proc. Natl. Acad. Sci. USA, 106, 19011–19016. 19887634 10.1073/pnas.0905466106 PMC2772816

[bty818-B12] Pedregosa F. et al (2011) Scikit-learn: machine learning in Python. J. Machine Learn. Res., 12, 2825–2830.

[bty818-B13] Pérez-Hernández G., Noé F. (2016) Hierarchical time-lagged independent component analysis: computing slow modes and reaction coordinates for large molecular systems. J. Chem. Theory Comput., 12, 6118–6129. 27792332 10.1021/acs.jctc.6b00738

[bty818-B14] Pérez-Hernández G. et al (2013) Identification of slow molecular order parameters for markov model construction. J. Chem. Phys., 139, 015102. 23822324 10.1063/1.4811489

[bty818-B15] Plattner N., Noé F. (2015) Protein conformational plasticity and complex ligand-binding kinetics explored by atomistic simulations and markov models. Nat. Commun., 6, 7653. 26134632 10.1038/ncomms8653 PMC4506540

[bty818-B16] Preto J., Clementi C. (2014) Fast recovery of free energy landscapes via diffusion-map-directed molecular dynamics. Phys. Chem. Chem. Phys., 16, 19181–19191. 24955434 10.1039/c3cp54520b

[bty818-B17] Saeys Y. et al (2007) A review of feature selection techniques in bioinformatics. Bioinformatics, 23, 2507–2517. 17720704 10.1093/bioinformatics/btm344

[bty818-B18] Schaudinnus N. et al (2016) Global langevin model of multidimensional biomolecular dynamics. J. Chem. Phys., 145, 184114. 27846702 10.1063/1.4967341

[bty818-B19] Schrödinger L. (2010) The pymol molecular graphics system, version 1.3 r1. *Py-MOL, The PyMOL Molecular Graphics System, Version*, **1**.

[bty818-B20] Shaw D.E. et al (2009) Millisecond-scale molecular dynamics simulations on anton. In: *Proceedings of the Conference on High Performance Computing Networking, Storage and Analysis*. ACM, Portland, Oregon, USA, p. 39.

[bty818-B21] Stolzenberg S. (2014) Multi-scale computational studies of molecular mechanisms in the function of membrane-proteins in the family of neurotransmitter transporters. Phd Dissertation, Cornell University.

[bty818-B22] Stolzenberg S. et al (2015) Mechanism of the association between na+ binding and conformations at the intracellular gate in neurotransmitter: sodium symporters. J. Biol. Chem., 290, 13992–14003. 25869126 10.1074/jbc.M114.625343 PMC4447972

[bty818-B23] Stolzenberg S. et al (2016) Computational approaches to detect allosteric pathways in transmembrane molecular machines. Biochim. Biophys. Acta, 1878, 1652–1662. 10.1016/j.bbamem.2016.01.010 PMC4877268 26806157

[bty818-B24] Stone J.E. et al (2010) Gpu-accelerated molecular modeling coming of age. J. Molecular Graphics Model., 29, 116–125. 10.1016/j.jmgm.2010.06.010 PMC2934899 20675161

[bty818-B25] Wieczorek M. et al (2016) Mhc class ii complexes sample intermediate states along the peptide exchange pathway. Nat. Commun., 7, 13224. 27827392 10.1038/ncomms13224 PMC5105163

[bty818-B26] Wriggers W. et al (2009) Automated event detection and activity monitoring in long molecular dynamics simulations. J. Chem. Theory Comput., 5, 2595–2605. 26631775 10.1021/ct900229u

