# Supplementary material for: PySFD: comprehensive molecular insights from significant feature differences detected among many simulated ensembles
Source: Bioinformatics. 2018 Sep 21;35(9):1588–90. doi: 10.1093/bioinformatics/bty818 (PMC6499238; doi:10.1093/bioinformatics/bty818)
Supplement: Supplementary Data [file bty818_supp.zip › bty818-suppl_data/PySFD.SI.pdf]

# PySFD: Comprehensive Molecular Insights from Significant Feature Differences Detected among many Simulated Ensembles (Supplementary Information)

Sebastian Stolzenberg

Computational Molecular Biology Group,
Department of Mathematics and Computer Science,
Arnimallee 6, 14195 Berlin, Germany.

# 1 Supplementary Methods

Given a number of molecular input trajectories for each simulated state ensemble, PySFD detects and visualizes significant feature differences (SFDs) in three different stages (I-III, Fig. 1):

## 1.1 Feature Extraction (Fig. 1, I)

PySFD currently considers the following groups of feature types in form of classes derived from a *FeatureAgent* parent class (see Fig. 1):

- *SRF*, single residual features: (relative, i.e. normalized) solvent-accessibility surface areas (*SASA_sr*, *RSASA_sr*, [18]), $C_\alpha$ root-mean square fluctuations (*CA_RMSF_VMD*), dihedrals (backbone torsions $\phi$, $\psi$, $\omega$, and side-chain rotamers $\chi_{1-4}$), chemical shifts, and scalar couplings (*J3_HN-C'*, -$C_\beta$, and -$H_\alpha$).
- *PRF*, pairwise residual features: distances and (partial) correlations ([7]); for computational efficiency, each residue is represented by its corresponding $C_\alpha$ atom or *SRF* feature, so that distances and correlations are computed between $C_\alpha$ positions, dihedrals, or scalar couplings. Depending on the feature type (linear or circular), I compute either Pearson or circular correlations (i.e. $\frac{\sum_{k=1}^{n}\sin(x_k-\tilde{x})\sin(y_k-\tilde{y})}{\sqrt{\sum_{k=1}^{n}\sin^2(x_k-\tilde{x})\sum_{k=1}^{n}\sin^2(y_k-\tilde{y})}}$, [4], for circular samples $x = (x_0, x_1, ..., x_n)$, $y = (y_0, y_1, ..., y_n)$ and circular means $\tilde{x}$ and $\tilde{y}$).
- *sPBSF*, sparse pairwise backbone/side-chain features:
  - non-covalent contact frequencies capturing the relative number of frames in a simulated ensemble, in which the atoms of a residual backbone or side-chain form a contact with the atoms of another residual backbone or side-chain (hydrogen bond, *HBond_...*, [9, 3, 11], or heavy-atom van der Waals radius contact, *Hvvdwdist_VMD*, [19], or both, *HvvdwdistHB*, [17]), and

  - non-covalent contact dwell times defined as the average simulation times a contact is formed ($\bar{t}_{on}$) or not formed ($\bar{t}_{off}$), respectively, until it is subsequently broken or formed; dwell times are currently implemented only for time-dependent input simulations, and from first tests, I observed that any significant difference in these dwell times could already be explained by significant differences in contact frequency (not shown).
  - These features are "sparse" because contacts are only tabulated, if they actually form in at least one simulated input frame.
  - *Hvvdwdist_VMD* contacts can be transformed into a single backbone/side-chain feature, e.g., by only considering the contact frequencies of any residual backbone or side-chain with water (*Hvvdwdist_H2O*, see Fig. S4.a).

- *PPRF*, pairwise pairwise residual features: (partial) correlations between *PRF* features (currently, only $C_\alpha$ distances).

- *PsPBSF*, pairwise sparse pairwise backbone/side-chain features, i.e. (partial) correlations between *sPBSF* features.

In each simulation frame, features are tabulated as Python pandas data frames ([13]) and can be further coarse-grained into user-defined regions by residual identity (and optionally by backbone/side-chain identity) via a user-defined function (e.g., mean or sum). Depending on the user's need towards computational platform-independence, speed, or accuracy, these features can be extracted directly within Python via the MDTraj module ([11]), or via external programs such as VMD ([3]), or HBPLUS ([9]). All these features can be processed simultaneously on multiple CPUs (using the Pathos module, [12]), so that *n* simulated ensembles and *n*m* underlying input simulations (replica) can be processed at the same time.

## 1.2 Computing Feature Type Redundancies (Fig. 1,II)

Currently, our PySFD implementation includes at least 18 feature types, and many more can be incorporated in the future. Therefore, it will become necessary to detect and possibly avoid redundancies between existing and newly implemented feature types. To this end, I have implemented *featuretype_redundancies,* a PySFD method which computes correlation coefficients ("pearson", "kendall", "spearman", or "circular") between feature types along individual feature labels (i.e. residue identity for *SRF* features) and ensemble means (i.e. means over ensemble trajectories - this is much more efficient than computing correlations over all sampled ensemble frames). To treat periodic boundaries of circular features for "pearson", "kendall", and "spearman" correlations (e.g., for combined data sets of both linear and circular feature types), I shift a particular circular data set by 360° with modulo 360°, if this shift results in a reduced variance (in PySFD, circular variables are originally measured between -180° and 180°). This is a simple, yet practically efficient strategy to compute linear-to-linear, circular-to-circular, and linear-to-circular correlation coefficients at the same time with a common correlation measure (as in Fig. S4.a). However, there exist more sophisticated strategies to compute such correlations (via multiple correlation coefficients, [5]), which may be implemented in the future. As a current default, I define two feature types to be redundant, if they share a monotonic correlation, i.e. an absolute Spearman correlation coefficient, of at least 0.75.

## 1.3 Computing SFDs (Fig. 1, II)

A statistical distribution with random variable $X$ on a compact support is uniquely characterized by its statistical moments $m^i = \langle X^i \rangle$ with ordinal $i \in \mathbb{N}$ [1]. Therefore, I consider a sampled

feature distribution to be significantly different between pairs of different simulated ensembles $a$ and $b$, if any of their statistical moments $m_a^i$, $m_b^i$ differ significantly, i.e. if

$$c_s^i \equiv \text{abs}\left(\bar{m}_a^i - \bar{m}_b^i\right) - \max\left(n_\sigma \cdot \sqrt{\Delta^2_{m_a^i} + \Delta^2_{m_b^i}}, n_f\right) > 0$$

with significant feature difference $d_s^i \equiv \text{sign}\left(\bar{m}_a^i - \bar{m}_b^i\right) \cdot c_s^i \neq 0$, where $\bar{m}_a^i$ and $\bar{m}_b^i$ are means over ensemble trajectories (i.e. not just feature trajectory means $m_a^1$, $m_b^1$ as in [16, 8]) with uncertainties $\Delta_{m_a^i}$, $\Delta_{m_b^i}$ and user-defined significance parameters $n_\sigma$ and $n_f$. $\Delta_{m_a^i}$ and $\Delta_{m_b^i}$ are defined either "statistically", i.e. either as a standard error ($\sigma_{\bar{m}_a^i}$, i.e. a standard deviation over ensemble trajectory means), or "effectively", i.e. a mean of standard deviations over ensemble trajectories. The names for these uncertainties are motivated by their resulting criteria for significance ($c_s$), which are somewhat equivalent to statistical significance testing (using two-sided Z-scores) and effect sizes ([6]), respectively. For this purpose, the advantage of using $c_s$ over Z-scores and effect sizes is that it results in the significant feature difference $d_s$, which can be directly interpreted in units of the particular feature type; for any "$c_s > 0$"-significant difference, however, PySFD also reports Z-scores/p-values and effect sizes. Under the assumptions of the central limit theorem, "statistical" uncertainties with $n_f = 0$ are valid for any underlying finite-variance distribution (which inherently was assumed to be normal in the previous PIA ([16]) and pyHVis3D ([8]) tools), whereas "effective" uncertainties make sense in the limit where a feature's distributions in two ensembles *a* and *b* are well-sampled and statistically significantly different, yet so broad, as reflected by large standard deviations in *a* and *b*, that from an effect size perspective the overlap between their underlying distributions is too large for them to be considered significantly different.

To actually test for a feature's significant difference, I implemented the following algorithm into PySFD: Iteratively, for each $n = 1,\ 2,\ ...,\ maxmomord$ ($maxmomord$ is a user-defined parameter), PySFD identifies any SFD in the $n^{\text{th}}$ central moment, that is not significantly different for all other central moments with ordinal $m < n$. In other words, PySFD first scans for any SFD in the mean ($n = 1$), then for any significant difference in the variance ($n = 2$), that are not significantly different in the mean, then for any significant difference in the third central moment, that are significantly different in neither the mean nor the variance, and so forth. PySFD then allows the user to verify the features identified to be significantly different by generating mean feature histograms from the individual ensemble trajectory feature histograms.

The ensemble trajectories mentioned above (DCD or XTC format) are either taken directly from frame input sample batches (i.e. trajectories each containing frames sampled from a stationary distribution of, e.g., a trajectory-bootstrapped MSM or a bayesian MSM sample), or from trajectory bootstraps of regular, molecular (dynamics) simulations.

PySFD also allows the identification of SFDs that are *common* among different ensemble comparisons, and optionally *not* significantly different among a different set of ensemble comparisons (e.g., see Fig. S1 and below). Such common ensemble differences may assist, e.g., to identify potential mechanistic elements that universally act as triggering "switches" in the function of a simulated protein.

## 1.4 Visualizing SFDs (Fig. 1, III)

PySFD currently contains separate VMD ([3]) and PyMOL ([15]) scripts that visualize (common) SFDs, as demonstrated below. A PySFD user can automatically access these PyMOL scripts by calling the *pysfd.view_feature_diffs()* method (see code documentation), which uses the module *iPyMol (https://github.com/mwojcikowski/iPyMol.git)*, in essence a PyMOL API. Other means for molecular visualization I plan to implement as they become available.

# 2 Supplementary Results and Discussion

To illustrate its functionality, I employed PySFD to analyze MSMs of an aggregate of 300$\mu s$ MD simulations I had recently performed ([21]) on a major histocompatibility complex class II (MHCII) protein ([20]): in the adaptive immune system, this peptide-exchanging protein is responsible for the correct recognition of antigenic peptides and their presentation to T-cell receptors. In experimental collaboration with experts in X-ray crystallography and NMR ([21]), I had simulated two point mutants ($\alpha$T41A and $\beta$N82A) with respect to a wild-type member of the MHCII family (HLA-DR1) in order to investigate the onsets of its catalyzed and uncatalyzed pathways of peptide exchange. Our major finding was that MHCII spontaneously samples intermediate conformations along these pathways, with $\alpha$T41A known to enhance peptide exchange along the catalyzed [22], and $\beta$N82A majorly along the uncatalyzed pathway (e.g., [10]). Though the molecular mechanisms along either of these pathways are probably distinct, they may share common elements that are potentially universal to peptide exchange.

## 2.1 (Common) SFDs

In view of such elements as common changes in hydrogen bonds (feature class *HBond_mdtraj*, Fig. 1), I first identified significant differences in hydrogen bond frequency for illustration between the MSM meta-stable ground states MS3$^{\alpha\text{T41A}}$ vs. MS3$^{\text{WT}}$ and between MS3$^{\beta\text{N82A}}$ vs. MS3$^{\text{WT}}$ (as tabulated in Files S1 and S2, also see Section 2.5; each meta-stable state is represented by 50 ensemble trajectories, each containing 100 simulation frames sampled from this meta-stable state, [21]). In Fig. S1, left and right panel, these differences are mapped onto snapshots of MS3$^{\beta\text{N82A}}$ and MS3$^{\alpha\text{T41A}}$, respectively, as orange (green) bars, if a hydrogen bond is significantly more (less) frequent in MS3$^{\beta\text{N82A}}$/MS3$^{\alpha\text{T41A}}$ than in MS3$^{\text{WT}}$. These bars form networks of alternating differences in hydrogen bonds (i.e. with alternating signs), which can extend across the entire peptide binding groove of MHCII: For example, the simulated $\beta$N82A mutant induces an alternating difference network in MS3$^{\beta\text{N82A}}$ vs. MS3$^{\text{WT}}$ (Fig. 1, left) that extends from the site of mutation ($\beta$N82A, i.e. at peptide binding pocket P2) via pockets P4 and P5 all the way to pocket P10 (binding pockets are labeled as in [14], i.e. P-1 with representative residue $\beta$His81; P4: $\alpha$Gln9, $\alpha$Asn62; P5: $\beta$Arg71, P10: $\alpha$Arg71, $\beta$Asp57). Such alternating networks thus reflect molecular couplings connecting different peptide binding pockets along the entire binding groove, which have been inferred previously ([23, 21, 20]). Among the significant hydrogen bond differences shown in Fig. S1, the ones between $\alpha$Hip33 and $\alpha$Glu40 and between $\alpha$Asn62 and $\chi$Ala110 share the same sign between MS3$^{\beta\text{N82A}}$ vs. MS3$^{\text{WT}}$ and MS3$^{\alpha\text{T41A}}$ vs. MS3$^{\text{WT}}$, as highlighted in Fig. S1, central panel. The residues involved in these hydrogen bonds are not limited to the binding groove, are conserved among the MHCII family, and may thus act as common mechanistic switches in both the catalyzed and uncatalyzed pathway of peptide exchange.

## 2.2 Coarse-graining Features

PySFD's general capability to coarse-grain features into structural regions is illustrated in Fig. S2: Here, I have identified significantly longer (shorter) $C_\alpha$-to-$C_\alpha$ distances (colored proportionally to their significant difference $d_s$) in the intermediate MS1$^{\beta\text{N82A}}$ with respect to the ground state MS3$^{\text{WT}}$ as orange (green) bars on a snapshot of MS1$^{\beta\text{N82A}}$. In the right panel, I have coarse-grained the $C_\alpha$ atoms into structural regions (triplets of consecutive residues), which allows a representation of overall distance differences with much fewer bars. Taken together, these significant distance differences characterize the MS1$^{\beta\text{N82A}}$ intermediate with respect to MS3$^{\text{WT}}$ by a tighter packing (green bars) between the CLIP peptide (black)/the DM-binding site of the

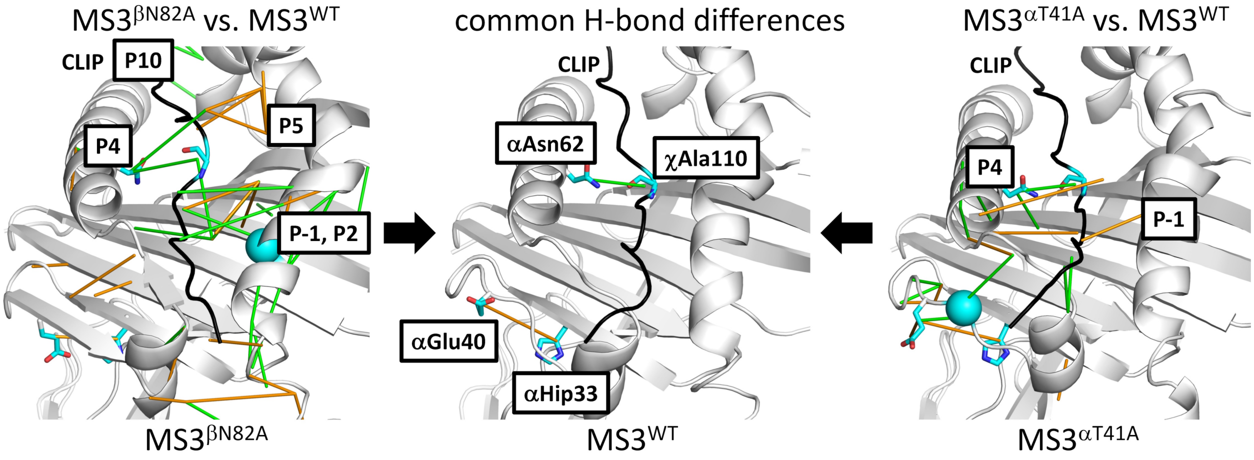


| donor | acceptor | WT | bN82A | aT41A |
|---|---|---|---|---|
| αHip33 (sidechain) | αGlu40 (sidechain) | 0.01 ± 0.01 | 0.09 ± 0.03 | 0.09 ± 0.03 |
| αAsn62 (sidechain) | χAla110 (backbone) | 0.65 ± 0.05 | 0.46 ± 0.06 | 0.41 ± 0.06 |

Figure S1: **(Common) Significant differences in hydrogen bond frequencies** between MHCII's ground states of the $\beta$N82A and $\alpha$T41A mutants with respect to wild-type (WT). Shown are white ribbon representations of snapshots of these ground states (MS3) of $\beta$N82A (left), $\alpha$T41A (right), and WT (center) viewed into the binding groove that is occupied with the CLIP peptide (black); the respective points of mutation for $\beta$N82A and $\alpha$T41A are indicated by cyan spheres. Orange and green bars respectively represent hydrogen bonds ("*HBond_mdtraj*", $n_\sigma = 2$ and $n_f = 0$, "statistical" uncertainties, see Methods section) which are significantly more and less frequent in $\beta$N82A/$\alpha$T41A with respect to WT. These bars are drawn between the centers of mass of two hydrogen bond partners (residual side-chain or backbone); the frequencies of these hydrogen bonds are listed in Files S1 and S2. Peptide binding pockets (P-1, P2, ..., P10) of MHCII involved in these significant hydrogen bond differences are labeled as in ([14]) (see text). Among these significantly different hydrogen bonds, two between $\alpha$Hip33 and $\alpha$Glu40 and between $\alpha$Asn62 and $\chi$Ala110 ($\chi$ indicates CLIP) are significantly more and less frequent, respectively, in both mutants compared to WT, as tabulated below the panels. The residues involved in these hydrogen bonds are indicated as sticks in all three snapshots, and highlighted in the central panel ("common H-bond differences"). The two black arrows represent a merge of the two ensemble comparisons into a simplified representation of commonly significant differences in hydrogen bond frequencies. This figure was rendered with PyMOL.

a) Peptide association (i.e. "Hvvdwdist_VMD" contact with MHCII)

| Residue ID | backbone (b) / side-chain (s) | bN82A | WT | ds |
|---|---|---|---|---|
| γArg108 | b | 0.89 ± 0.05 | 1.00 ± 0.00 | -0.005 |
| γArg108 | s | 0.73 ± 0.08 | 0.99 ± 0.01 | -0.107 |
| γMet109 | b | 0.81 ± 0.07 | 0.98 ± 0.03 | -0.018 |
| γAla110 | b | 0.83 ± 0.06 | 0.99 ± 0.02 | -0.044 |

b) Binding groove hydration (i.e. “Hvvdwdist_VMD“ contact with water)

| Residue ID | backbone (b) / side-chain (s) | bN82A | WT | ds |
|---|---|---|---|---|
| αIle7 | b | 0.29 ± 0.13 | 0.81 ± 0.05 | -0.239 |
| αIle7 | s | 0.13 ± 0.06 | 0.64 ± 0.16 | -0.175 |
| αIle8 | b | 0.20 ± 0.09 | 0.48 ± 0.11 | -0.003 |
| αGln9 | s | 0.54 ± 0.17 | 0.14 ± 0.05 | 0.038 |
| αPhe24 | s | 0.14 ± 0.07 | 0.44 ± 0.10 | -0.059 |
| βPhe13 | b | 0.37 ± 0.10 | 0.77 ± 0.06 | -0.166 |
| βThr77 | b | 0.90 ± 0.05 | 0.73 ± 0.05 | 0.020 |
| βTyr78 | b | 0.49 ± 0.09 | 0.25 ± 0.06 | 0.036 |
| βTyr78 | s | 0.83 ± 0.06 | 0.97 ± 0.03 | -0.010 |
| βN82A | b | 0.52 ± 0.09 | 0.20 ± 0.07 | 0.089 |
| βN82A | s | 0.31 ± 0.07 | 0.88 ± 0.10 | -0.315 |
| γTrp107 | s | 0.72 ± 0.11 | 0.08 ± 0.06 | 0.396 |
| γLeu113 | s | 0.73 ± 0.08 | 0.92 ± 0.04 | -0.014 |

Table S1: **Significant differences in MHCII's peptide association and binding groove hydration induced by the simulated βN82A mutation (in reference to Fig. S1).** a) Peptide association: Heavy atom contact frequencies (± standard errors, n = 2, nf = 0) between MHCII and any residual peptide backbone or side-chain are listed with significant differences ds. Negative values of ds are colored in green (i.e. if a frequency is significantly higher in MS3N82A than in MS3WT). b) Binding groove hydration: Contact frequencies (as above) between water and any residual backbone or side-chain of MHCII or peptide. Positive (negative) values of ds are colored in blue (red), i.e. if a frequency is significantly higher in MS3N82A than in MS3WT. Peptide association (i.e. “Hvvdwdist_VMD“ contact with MHCII).

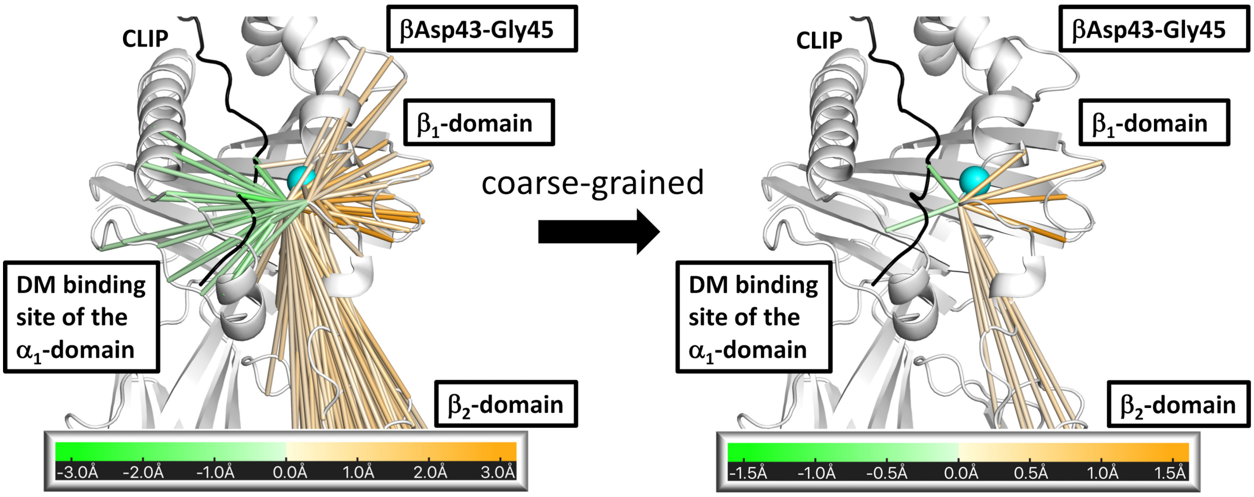


Figure S2: **(Coarse-grained) significant differences in C$_\alpha$-to-C$_\alpha$ distances** between MS1$^{\beta\text{N82A}}$ and MS3$^{\text{WT}}$ are mapped in each panel onto a snapshot of MS1$^{\beta\text{N82A}}$, as represented in Fig. S1: Orange and green bars respectively represent C$_\alpha$-to-C$_\alpha$ distances ($n_\sigma = 2$ and $n_f = 0$, "effective" uncertainties, see Methods section) which are significantly longer and shorter in MS1$^{\beta\text{N82A}}$ compared to MS3$^{\text{WT}}$; these bars are color-coded by their significant feature differences $d_s$ as seen by a color ramp in each panel. On the left, significant differences of all C$_\alpha$-to-C$_\alpha$ distances are shown, whereas on the right, such significant differences are coarse-grained distances, i.e., average C$_\alpha$-to-C$_\alpha$ distances among triplets of residues along the protein sequence of MHCII. This figure was rendered with PyMOL.

$\alpha_1$-domain and the broken $\alpha$-helix of the $\beta_1$ domain (cyan sphere), and a widening (orange bars) between this broken $\alpha$-helix and other regions of the $\beta$ domain (loops of the $\beta_1$ domain, regions of the $\beta_2$ domain). The fact that, e.g., the four significantly different C$_\alpha$-to-C$_\alpha$ distances with individual residues of the triplet $\beta$Asp43-Gly45 are not significantly different after coarse-graining (see orange bars in Fig. S2) is due to triplet averaging: in fact, these distances are between $\beta$Asp43/$\beta$Tyr83, $\beta$Asp43/$\beta$Gly84, $\beta$Val44/$\beta$Tyr83, and $\beta$Gly45/$\beta$Tyr83, so that after triplet averaging between the triplets $\beta$Asp43-Gly45 and $\beta$N82A-Gly84, these individual significant differences get smeared out. This artifact exemplifies the need for a careful coarse-graining definition for a particular simulated system under study, which has been omitted here for demonstration purposes.

Such careful coarse-graining definitions, on the other hand, can provide substantial mechanistic insights into a simulated molecule, as rendered in Fig. S3: Here, PySFD has selected significant differences in *Hvvdwdist_VMD* contact frequencies in MS3$^{\beta\text{N82A}}$ with respect to MS3$^{\text{WT}}$ between (i) MHCII and any residual peptide backbone or side-chain (rendered as green sticks for significantly lower frequencies) and (ii) between water and any residual backbone or side-chain of MHCII or peptide (rendered as blue/red sticks for significantly higher/lower frequencies); the frequencies of these contacts are listed in Table S1. As a result, Fig. S3 illustrates the partial dissociation of the CLIP peptide previously observed in MS3$^{\beta\text{N82A}}$ ([21]), which PySFD finds to coincide with heterogeneous changes in hydration of MHCII's peptide binding groove. As the $\beta$N82A mutant promotes the uncatalyzed pathway of MHCII's peptide exchange, this finding characterizes MS3$^{\beta\text{N82A}}$ and thus the onset of uncatalyzed exchange with distinct conformational rearrangements in the binding groove, exemplifying the importance of key mechanistic features automatically inferred and visualized by PySFD.

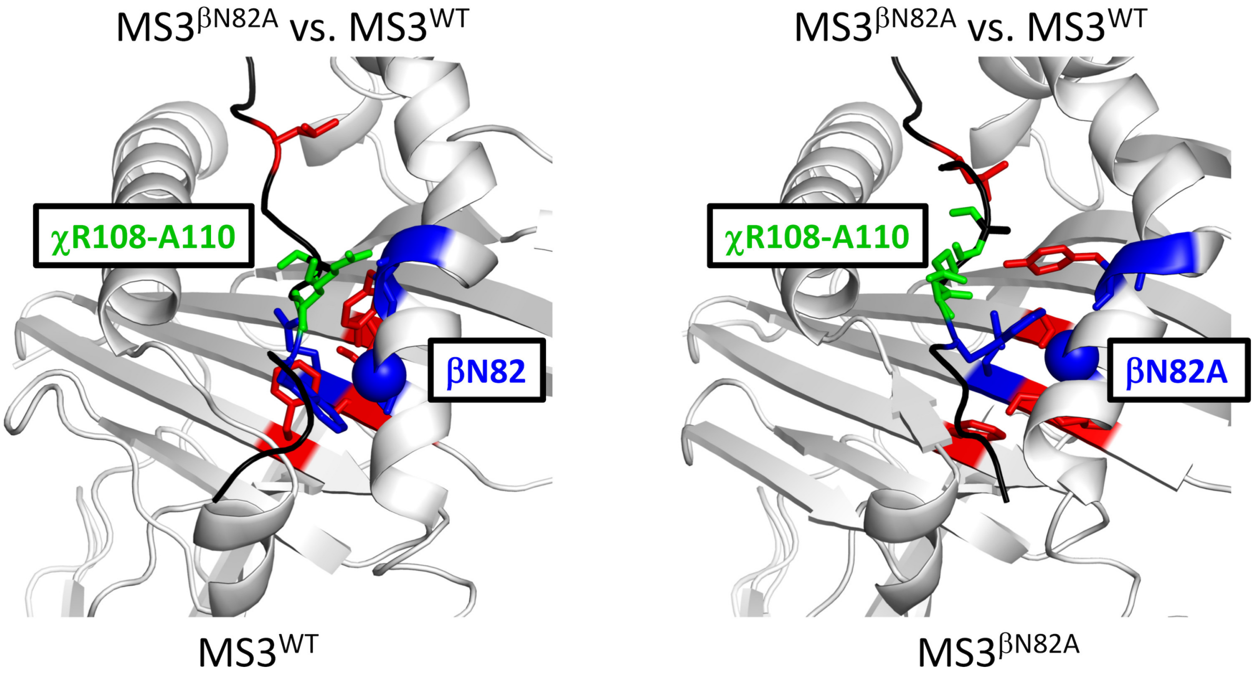


Figure S3: **Significant differences in peptide association and binding groove hydration induced by the** $\beta$N82A **mutation in MHCII.** A snapshot of the simulated MS3$^{\beta \text{N82A}}$ state is represented as in Fig. S1 and overlaid with significant differences between MS3$^{\beta \text{N82A}}$ and MS3$^{\text{WT}}$ ($n_\sigma = 2$, $n_f = 0$, "statistical" uncertainties, see Methods section) in heavy atom contact frequencies (*Hvvdwdist_VMD*) between MHCII and any residual peptide backbone or side-chain (rendered as green sticks for significantly lower frequencies) and between water and any residual backbone or side-chain of MHCII or peptide (rendered as blue/red sticks for significantly higher/lower frequencies); some of the residues involved in these contacts are labeled for clarity, the frequencies of these contacts are listed in Table S1. This figure was rendered with PyMOL.

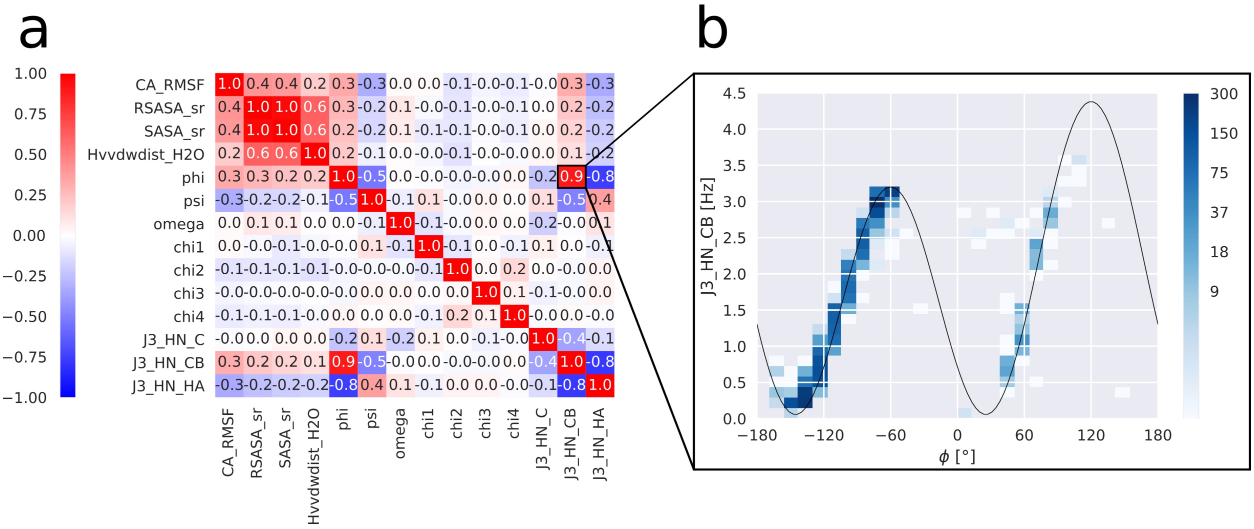


Figure S4: **Potential feature type redundancies detected with PySFD.** In (a), a matrix heat map shows Spearman correlation coefficients between *SRF* feature types along individual residues and ensemble means of all three meta-stable states of each simulated WT, $\beta$N82A, and $\alpha$T41A construct of MHCII. In (b), a heat map with logarithmic histogram coloring of sampled $^3\text{J}_{\text{HN-C}_\beta}$ vs. $\phi$ values is overlaid with a black plot of the Karplus relation $^3\text{J}_{\text{HN-C}_\beta}$ vs. $\phi$ ([2, 11]).

## 2.3 Detecting Feature Type Redundancies

As more feature types will be become available to PySFD, the more it will become necessary to avoid possible redundancies between new and existing feature types (and thus computational effort in the analysis). For the *SRF* feature type group, I could already identify potential feature type redundancies (see Supplementary Methods section), e.g., between the $^3\text{J}_{\text{HN-C}_\beta}$ scalar coupling and the $\phi$ backbone dihedral (Fig. S4.a) with a Spearman correlation coefficient of 0.9 over individual residues and ensemble means of all three meta-stable states of each simulated WT, $\beta$N82A, and $\alpha$T41A construct of MHCII. At first sight, this similar-to-monotonic correlation is surprising because the corresponding Karplus relationship reads $^3\text{J}_{\text{HN-C}_\beta}(\phi) = 3.71 \cdot \cos^2(\phi + 60°) - 0.59 \cdot \cos(\phi + 60°) + 0.08$ Hz ([2, 11]), as plotted in black in Fig. S4.b. However, our MD simulations mostly sample negative $\phi$ values (consistent with a usual $\psi/\phi$ Ramashandran plot for proteins), for which $^3\text{J}_{\text{HN-C}_\beta}$ grows almost linearly with $\phi$ (heat map in Fig. S4.b).

Therefore, this result demonstrates how two feature types that appear monotonically uncorrelated along their mathematically defined ranges can in fact be correlated, as the underlying MD-simulations of the protein may only sample a subset of these values with non-uniform weights. In general, there may not even exist a clearly defined relation between two feature types (as for $^3\text{J}_{\text{HN-C}_\beta}(\phi)$), which in this case can only be revealed empirically from MD simulations, e.g., via PySFD.

## 2.4 Detecting Spurious Significances of Feature Differences

As a consequence of the well-known sampling problem of MD simulations, significant feature differences detected by PySFD are prone to deception, as illustrated by the following example: In Fig. S5, I compute average numbers of significantly different $C_\alpha$-to-$C_\alpha$ distances both with "statistical" (a) and "effective" (b) uncertainties ($n_\sigma = 1$, $n_f = 0$, see Supplementary Methods) between $n_b$ bootstrapped MHCII mutant ($\beta$N82A) and $n_b$ wild type simulations [20] and plot these average numbers against $n_b$. Here, each average is plotted with its standard error as taken over 200 bootstraps (with replacement), each with varying value of $n_b$. Plotted are average numbers for either all SFDs ("all SFDs"), the intersection of all SFDs with the ones of the

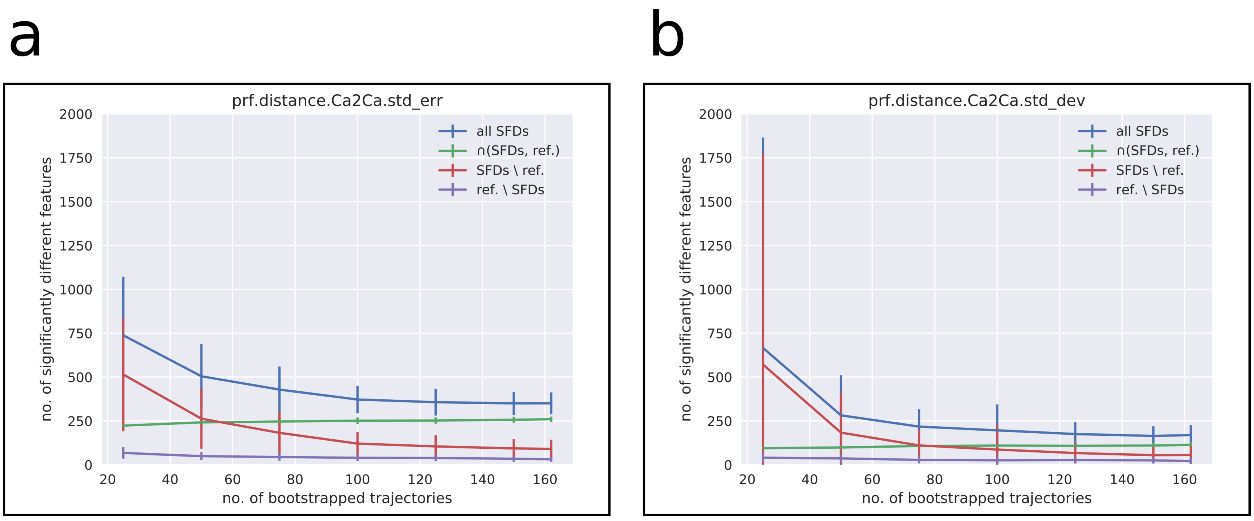


Figure S5: **Lack of sampling may result in spurious significances of feature differences.** Average number of significantly different features ($C_\alpha$-to-$C_\alpha$ distances, with "statistical" (a) and "effective" (b) uncertainties, see Supplementary Methods section) between the MHCII mutant ($\beta$N82A) and wild type simulations [20] are plotted against the number of bootstrapped trajectories. Each average is plotted with its standard error and taken over 100 bootstraps (with replacement), each with varying numbers of bootstrapped trajectories. Plotted are average numbers for either all SFDs ("all SFDs"), for the intersection of all SFDs with the ones of the reference simulation set ("∩(SFDs, ref."), i.e. all 2x162 MD simulation à 500-600ns [20]), for all SFDs that are not a reference SFD ("SFDs\ref."), and for all reference SFDs that are an bootstrapped SFDs.

reference simulation set ("∩(SFDs, ref.)", i.e. all 2x162 wild type and mutant MD simulations à 500-600ns [20]), for all SFDs that are not a reference SFD ("SFDs\ref."), and for all reference SFDs that are not a bootstrapped SFDs ("ref.\SFDs"). As seen in both panels, the total ("all SFDs") and surplus ("SFDs\ref.") numbers of SFDs on average decrease with increasing number of bootstrapped trajectories, $n_b$, converging at around $n_b = 100$, which indicates the existence of spurious SFDs for undersampled simulations. On the other hand, the numbers of intersecting ("∩(SFDs, ref.") and missing SFDs ("ref.\SFDs") stay fairly constant at a relatively higher and lower value along $n_b$, respectively. This finding indicates that for $n_b < 100$, these spurious SFDs resulting from a lack of sampling tend to increase the variance, but not the bias with respect to the converged SFD set - in other words, "true" (converged) SFDs tend to be SFDs also for $n_b < 100$. Within PySFD, the user can identify such spurious SFDs in a similar fashion as performed in Fig. S5, with the parameter $intrajdatatype$ = "convcheck" (see code documentation).

## 2.5 Supplementary Files S1 and S2 (in reference to Fig. 2)

In these files, significant differences in hydrogen bond frequencies between MHCII's ground states of the βN82A and αT41A mutants with respect to wild-type (WT):

- S1.spbsf.HBond_mdtraj.std_err.aT41A_vs_WT.MS3.csv
- S2.spbsf.HBond_mdtraj.std_err.bN82A_vs_WT.MS3.csv

Legend:

| | |
|---|---|
| seg1 (seg2) | segment/chain ID of donor (acceptor), "A", "B": MHCII, "c": CLIP peptide |
| res1 (res2) | residue ID of donor (acceptor) |
| rnm1 (rnm2) | residue (three letter) name of donor (acceptor) |
| bb1 (bb2) | 1, if backbone, 0 if otherwise of donor (acceptor) |
| mf | feature mean (here: *HBond_mdtraj* contact frequency) |
| sf | feature uncertainty (here: standard error) |
| sdiff | significant difference ds (ns = 2, nf = 0) |
| Zscore | corresponding Z score given "mf" and "sf" |
| pval | corresponding p-value given "mf" and "sf" |

# References


[1] Felix Hausdorff. Summationsmethoden und momentfolgen. *Mathematische Zeitschrift 9*, 1921.

[2] Jin-Shan Hu and Ad Bax. Determination of $\phi$ and $\chi1$ angles in proteins from 13c- 13c three-bond j couplings measured by three-dimensional heteronuclear nmr. how planar is the peptide bond? *Journal of the American Chemical Society*, 119(27):6360–6368, 1997.

[3] William Humphrey, Andrew Dalke, and Klaus Schulten. Vmd: Visual molecular dynamics. *Journal of Molecular Graphics*, 14(1):33–38, 1996.

[4] S Rao Jammalamadaka and Ashis Sengupta. *Topics In Circular Statistics. Series on Multivariate Analysis.* World Scientific, 2001.

[5] Richard A Johnson and Thomas Wehrly. Measures and models for angular correlation and angular-linear correlation. *Journal of the Royal Statistical Society. Series B (Methodological)*, pages 222–229, 1977.

[6] Ken Kelley and Kristopher J Preacher. On effect size. *Psychological methods*, 17(2):137, 2012.

[7] Seongho Kim. ppcor: an r package for a fast calculation to semi-partial correlation coefficients. *Communications for statistical applications and methods*, 22(6):665, 2015.

[8] Bernhard Knapp, Marta Alcala, Hao Zhang, Clare West, P Anton van der Merwe, and Charlotte M Deane. pyhvis3d: Visualising molecular simulation deduced h-bond networks in 3d: Application to t-cell receptor interactions. *Bioinformatics*, 1:3, 2018.

[9] IK McDonald, D Naylor, D Jones, and JM Thornton. Hbplus computer program. *Department of Biochemistry and Molecular Biology, University College, London, UK*, 1993.

[10] Benjamin J McFarland, John F Katz, Craig Beeson, and Andrea J Sant. Energetic asymmetry among hydrogen bonds in mhc class ii peptide complexes. *PNAS*, 98(16):9231–9236, 2001.

[11] Robert T. McGibbon, Kyle A. Beauchamp, Matthew P. Harrigan, Christoph Klein, Jason M. Swails, Carlos X. Hernández, Christian R. Schwantes, Lee-Ping Wang, Thomas J. Lane, and Vijay S. Pande. Mdtraj: A modern open library for the analysis of molecular dynamics trajectories. *Biophysical Journal*, 109(8):1528 – 1532, 2015.

[12] Michael M McKerns, Leif Strand, Tim Sullivan, Alta Fang, and Michael AG Aivazis. Building a framework for predictive science. *arXiv preprint arXiv:1202.1056*, 2012.

[13] Wes McKinney. Data structures for statistical computing in python. In Stéfan van der Walt and Jarrod Millman, editors, *Proceedings of the 9th Python in Science Conference*, pages 51 – 56, 2010.

[14] Corrie A Painter and Lawrence J Stern. Conformational variation in structures of classical and non-classical mhcii proteins and functional implications. *Immunological Reviews*, 250(1):144–157, 2012.

[15] LLC Schrödinger. The pymol molecular graphics system, version 1.3 r1. *Py-MOL, The PyMOL Molecular Graphics System, Version*, 1, 2010.

[16] Sebastian Stolzenberg, Mayako Michino, Michael V LeVine, Harel Weinstein, and Lei Shi. Computational approaches to detect allosteric pathways in transmembrane molecular machines. *Biochimica et Biophysica Acta (BBA)-Biomembranes*, 2016.

[17] Sebastian Stolzenberg, Matthias Quick, Chunfeng Zhao, Kamil Gotfryd, George Khelashvili, Ulrik Gether, Claus J. Loland, Jonathan A. Javitch, Sergei Noskov, Harel Weinstein, and Lei Shi. Mechanism of the association between na+ binding and conformations at the intracellular gate in neurotransmitter:sodium symporters. *Journal of Biological Chemistry*, 290(22):13992–14003, 2015.

[18] Matthew Z Tien, Austin G Meyer, Dariya K Sydykova, Stephanie J Spielman, and Claus O Wilke. Maximum allowed solvent accessibilites of residues in proteins. *PloS one*, 8(11):e80635, 2013.

[19] AJ Venkatakrishnan, Xavier Deupi, Guillaume Lebon, Christopher G Tate, Gebhard F Schertler, and M Madan Babu. Molecular signatures of g-protein-coupled receptors. *Nature*, 494(7436):185–194, 2013.

[20] Marek Wieczorek, Esam T Abualrous, Jana Sticht, Miguel Álvaro-Benito, Sebastian Stolzenberg, Frank Noé, and Christian Freund. Major histocompatibility complex (mhc) class i and mhc class ii proteins: Conformational plasticity in antigen presentation. *Frontiers in Immunology*, 8, 2017.

[21] Marek Wieczorek, Jana Sticht, Sebastian Stolzenberg, Sebastian Günther, Christoph Wehmeyer, Zeina El Habre, Miguel Alvaro-Benito, Frank Noé, and Christian Freund. Mhc class ii complexes sample intermediate states along the peptide exchange pathway. *Nature Communications*, 7, 2016.

[22] Liusong Yin and Lawrence J Stern. Hla-dm focuses on conformational flexibility around p1 pocket to catalyze peptide exchange. *Frontiers in immunology*, 4, 2013.

[23] Liusong Yin, Peter Trenh, Abigail Guce, Marek Wieczorek, Sascha Lange, Jana Sticht, Wei Jiang, Marissa Bylsma, Elizabeth D Mellins, Christian Freund, and Lawrence J Stern. Susceptibility to hla-dm protein is determined by a dynamic conformation of major histocompatibility complex class ii molecule bound with peptide. *Journal of Biological Chemistry*, 289(34):23449–23464, 2014.
